# LESSONS LEARNED ENGAGING CARE PARTNERS OF PEOPLE LIVING WITH DEMENTIA IN CO-DESIGN OF DIGITAL HEALTH INTERVENTIONS

**DOI:** 10.1093/geroni/igae098.2071

**Published:** 2024-12-31

**Authors:** Nicole Werner

**Affiliations:** Indiana University, Bloomington, Bloomington, Indiana, United States

## Abstract

Per the NIH Stage Model of Behavioral Intervention Development, once a scientific mechanism for a possible behavioral intervention is established, the next critical step is to systematically design the intervention with the target population. If digital health intervention researchers bypass this stage, their interventions may work in ideal but not “real world” settings. Moreover, partnerships between user-centered design/engineering experts and health sciences researchers are believed to “increase the likelihood for impactful outcomes from the use of the engineering design process.” However, more work is needed to translate user-centered design approaches to digital health intervention design for older adults and care partners. Co-design in particular promotes strengths-based (vs. deficit-based) treatment of older adults and care partners. That is, rather than being the focus of a study on what is wrong, older adults and their care partners actively contribute to design and are therefore more likely to produce digital health solutions that are usable, useful, relevant, acceptable, and adaptable to current routines. However, co-design approaches can be challenging to implement, and guidance for engaging older adults and care partners in co-design is lacking. Dr. Werner will present lessons learned across four projects that implemented a remote, five-phase co-design process to co-design digital health interventions with care partners of people living with dementia. Lessons learned span co-design activities, co-design quality, and co-designer outcomes that were identified from research team and care partner interviews conducted at the end of the design process and feedback surveys conducted with care partners after each co-design session.

